# Correction: Reconstructing the phase of vanadium oxides enables redox-catalysis manipulated reversible sulfur conversion for stable Zn–S batteries

**DOI:** 10.1039/d4sc90253j

**Published:** 2025-01-06

**Authors:** Hao Luo, Fan Li, Mingli Wang, Shang Sun, Min Zhou, Wenjing Zhang, Hengrui Guo, Xueyin Su, Xiaolong Li, Lina Ma

**Affiliations:** a School of Materials Science and Engineering, Xiamen University of Technology Xiamen 361024 China luohao_hit@163.com; b College of Chemistry and Chemical Engineering, Qingdao University Qingdao 266071 China malina@qdu.edu.cn; c State Key Laboratory of Polymer Materials Engineering, Polymer Research Institute, Sichuan University Chengdu 610065 P.R. China xiaolongli@scu.edu.cn; d School of Materials Science and Engineering, Beijing University of Chemical Technology Beijing 100029 China mingliw2000@163.com; e School of Materials Science and Engineering, Zhengzhou University Zhengzhou 450001 China

## Abstract

Correction for ‘Reconstructing the phase of vanadium oxides enables redox-catalysis manipulated reversible sulfur conversion for stable Zn–S batteries’ by Hao Luo *et al.*, *Chem. Sci.*, 2025, https://doi.org/10.1039/d4sc06593j.

The original version of Fig. 5c showed the S 2p XPS spectrum for the original state in the 1.5 V data. This has been corrected in the new Fig. 5 given below, which shows S characteristic peaks at 163.0 eV and 167.88 eV during charging at 1.5 V.

**Fig. 5:**
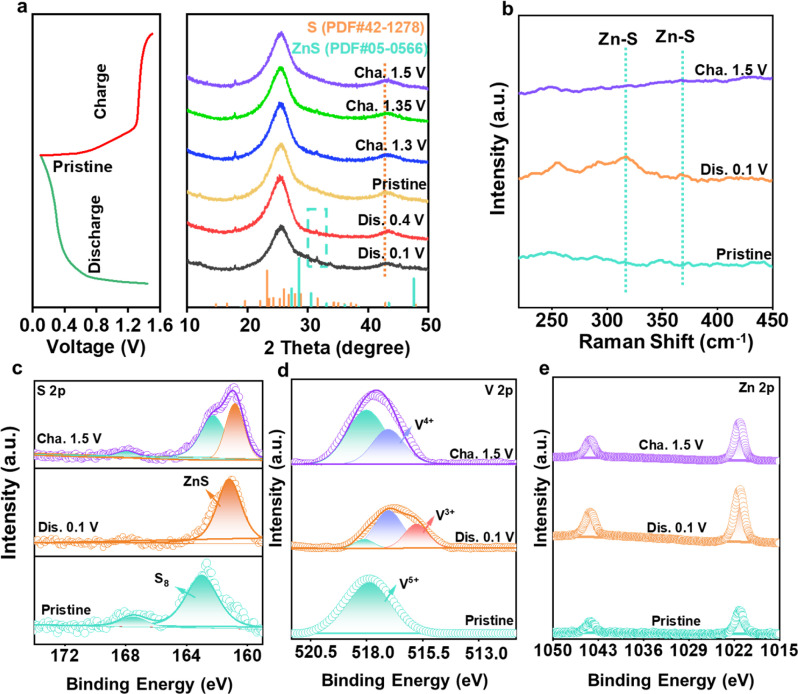
Analysis of reversible sulfur redox conversion. (a) *Ex situ* XRD patterns collected at different states. (b) Raman spectra at different charge and discharge states. XPS spectra of (c) S 2p, (d) V 2p, and (e) Zn 2p at different states.

The overall conclusions from the rest of the study remain unchanged.

The Royal Society of Chemistry apologises for these errors and any consequent inconvenience to authors and readers.

